# SWI/SNF and Asf1 Independently Promote Derepression of the DNA Damage Response Genes under Conditions of Replication Stress

**DOI:** 10.1371/journal.pone.0021633

**Published:** 2011-06-27

**Authors:** Laura V. Minard, Ling-ju Lin, Michael C. Schultz

**Affiliations:** Department of Biochemistry, School of Molecular and Systems Medicine, Faculty of Medicine and Dentistry, University of Alberta, Edmonton, Alberta, Canada; Texas A&M University, United States of America

## Abstract

The histone chaperone Asf1 and the chromatin remodeler SWI/SNF have been separately implicated in derepression of the DNA damage response (DDR) genes in yeast cells treated with genotoxins that cause replication interference. Using genetic and biochemical approaches, we have tested if derepression of the DDR genes in budding yeast involves functional interplay between Asf1 and SWI/SNF. We find that Asf1 and SWI/SNF are both recruited to DDR genes under replication stress triggered by hydroxyurea, and have detected a soluble complex that contains Asf1 and the Snf2 subunit of SWI/SNF. SWI/SNF recruitment to DDR genes however does not require Asf1, and deletion of Snf2 does not affect Asf1 occupancy of DDR gene promoters. A checkpoint engagement defect is sufficient to explain the synthetic effect of deletion of *ASF1* and *SNF2* on derepression of the DDR genes in hydroxyurea-treated cells. Collectively, our results show that the DDR genes fall into a class in which Asf1 and SWI/SNF independently control transcriptional induction.

## Introduction

SWI/SNF is a large multi-subunit enzyme that remodels chromatin by a mechanism requiring its DNA-stimulated ATPase activity [1]-[5]. In yeast, SWI/SNF contains 12 subunits [6], [7], including a conserved catalytic core made up of Snf2, Snf5 and Swi3 [8]. Snf2, the ATPase subunit of SWI/SNF, contributes to the regulation of approximately 6% of genes [9], [10]. At these genes, SWI/SNF alters histone-DNA interactions by promoting intra-nucleosomal DNA looping that leads to histone displacement. This activity underlies stimulation of transcription initiation and elongation by SWI/SNF [11]. SWI/SNF may also have roles in transcriptional repression [9], [10], [12] and the control of DNA replication [13], DNA silencing [14], and DNA repair [15], [16]. The function of SWI/SNF remains of great interest because it can function as a tumour suppressor in human cells [17], [18].

Like SWI/SNF, the non-enzymatic histone H3/H4 chaperone Asf1 also regulates the interactions of histones with DNA [19]-[22]. Asf1 can directly promote deposition of H3/H4 dimers onto DNA. By stimulating the activity of enzymes that acetylate H3 at K9/14, K27 and K56, Asf1 likely also indirectly affects the configuration of nucleosomes and higher-order chromatin structures [23]. The cellular functions that can involve SWI/SNF (transcription, silencing, DNA replication and repair) can also be modulated by Asf1. Therefore, although SWI/SNF and Asf1 control chromatin by fundamentally different mechanisms, they likely collaborate in the regulation of DNA-dependent processes that occur in the context of chromatin.

Microarray analysis of mRNA expression has revealed significant overlap between the genes affected by deletion of *ASF1* and the genes affected by deletion of components of SWI/SNF [9], [24]. These results raise the possibility that Asf1 and SWI/SNF have common functions in transcription. Mechanistic evidence in favour of this proposal has been obtained in studies of the *PHO5* and *HO* genes of budding yeast [12], [25]-[29]. At *PHO5*, SWI/SNF functions in advance of Asf1 to promote activator binding under low phosphate inducing conditions. Subsequent stable association of SWI/SNF with the *PHO5* promoter requires Asf1 [28]. At *HO*, SWI/SNF recruitment by activator bound to the URS1 upstream promoter element paves the way for Asf1 association at a downstream site, URS2. The latter event is necessary for SWI/SNF recruitment to the downstream URS2 element [25]. The evidence that *PHO5* and *HO* depend on Asf1 and SWI/SNF for induction, and that a SWI/SNF recruitment step at these genes depends on Asf1, strongly suggests an important role for Asf1 in SWI/SNF functions that affect transcription.

Asf1 and SWI/SNF have also been implicated in the regulation of two well-studied DNA damage response (DDR) genes, *RNR3* and *HUG1*. This regulation has been characterized in cells treated with genotoxins that inhibit replication: hydroxyurea (HU), which causes replication fork pausing by triggering depletion of deoxyribonucleoside triphosphates (dNTPs), and methane methylsulfonate (MMS), which modifies DNA in a way that causes fork blocking. Asf1 promotes derepression of *RNR3* and *HUG1* upon treatment with HU [26], and SWI/SNF is important for derepression of *RNR3* in cells treated with MMS [30]. These results set the stage for our experiments aimed at testing whether there is a direct functional interplay between Asf1 and SWI/SNF in transcriptional derepression of the DDR genes under conditions of replication stress triggered by HU.

## Results

### Derepression of two DDR genes under conditions of replication stress depends on Asf1 and SWI/SNF

**Figure 1:**
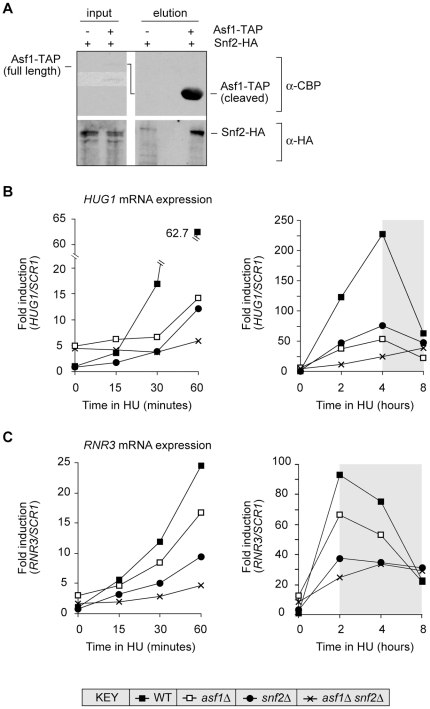
Asf1 and SWI/SNF: physical interaction and regulation of DDR gene transcription. A. Snf2 copurifies with Asf1. Asf1-TAP Snf2-HA and Snf2-HA lysates were used for tandem affinity purification. Inputs were obtained before binding to the first affinity column. Final eluates were resolved by SDS-PAGE and probed with either anti-CBP (top) or anti-HA (bottom) antibodies to detect Asf1 or Snf2, respectively. The high-contrast insert in the upper input panel has been included to more clearly show the presence of Asf1-TAP in the Asf1-TAP Snf2-HA lysate. B, C. Asf1 and SWI/SNF promote derepression of the DDR genes *HUG1* (B) and *RNR3* (C). Expression is normalised to *SCR1*. The grey shading in the right panel of B and C indicates the time period when *HUG1* and *RNR3* mRNA expression declines in the presence of HU.

Separate studies of Asf1 and SWI/SNF have revealed participation of both factors in transcriptional regulation of the DDR genes [26], [30]. We extended these studies by testing if Asf1 and Snf2 function together to regulate their derepression. This hypothesis was explored for two reasons. First, Asf1 and SWI/SNF may collaborate in the activation of *HO* [25]. Second, using a standard tandem affinity purification (TAP) protocol [31], we have detected a rare protein complex that includes Asf1 and Snf2. Specifically, we recovered Snf2 in a protein complex obtained by TAP from a strain which expresses Asf1-TAP and Snf2-HA (Figure 1A). Although only a minor fraction of Asf1 is associated with Snf2 in yeast, this finding suggests that the interaction between Asf1 and SWI/SNF first reported in *Drosophila* [32] may be more conserved in eukaryotes than previously appreciated.

We used Northern blotting to test for a possible functional interaction between Asf1 and SWI/SNF in the regulation of the DDR genes in yeast. Specifically, we measured mRNA expression of *HUG1* and *RNR3* in cells lacking *ASF1*, *SNF2*, or both, under conditions of replication stress triggered by growth in the presence of 0.2 M HU (the base medium for all experiments shown is YPD). Hydroxyurea treatment of wild type cells elicits robust derepression of *HUG1* and *RNR3* [33], [34], although these genes differ somewhat in their kinetics and fold of derepression. That is, *HUG1* is more strongly derepressed than *RNR3*, and its expression peaks approximately 2 hours after that of *RNR3* (right panels in Figure 1B, C). Note that the expression of *HUG1* and *RNR3* mRNA declines upon prolonged exposure to HU (grey shading, right panels in Figure 1B, C), and that mRNA down-regulation is initiated at different times at these genes: we have not studied the basis of this regulation.

Derepression of *HUG1* and *RNR3* occurred at a slower rate in the *asf1Δ* and *snf2Δ* single mutants (left panels in Figure 1B, C), raising the possibility that Asf1 and SWI/SNF are in a linear pathway that regulates transcription of the DDR genes. However, deletion of both *ASF1* and *SNF2* has a greater negative effect on derepression than deletion of either gene on its own (Figure 1B, C). Although Asf1 and Snf2 may coexist in the same complex, these transcription phenotypes suggest that Asf1 and SWI/SNF work separately to promote the rapid derepression of DDR genes when cells are cultured under conditions of replication stress. Several lines of evidence, described below, are consistent with this interpretation.

### Independent recruitment of Asf1 and Snf2 to DDR gene promoters during replication stress induced by HU

**Figure 2:**
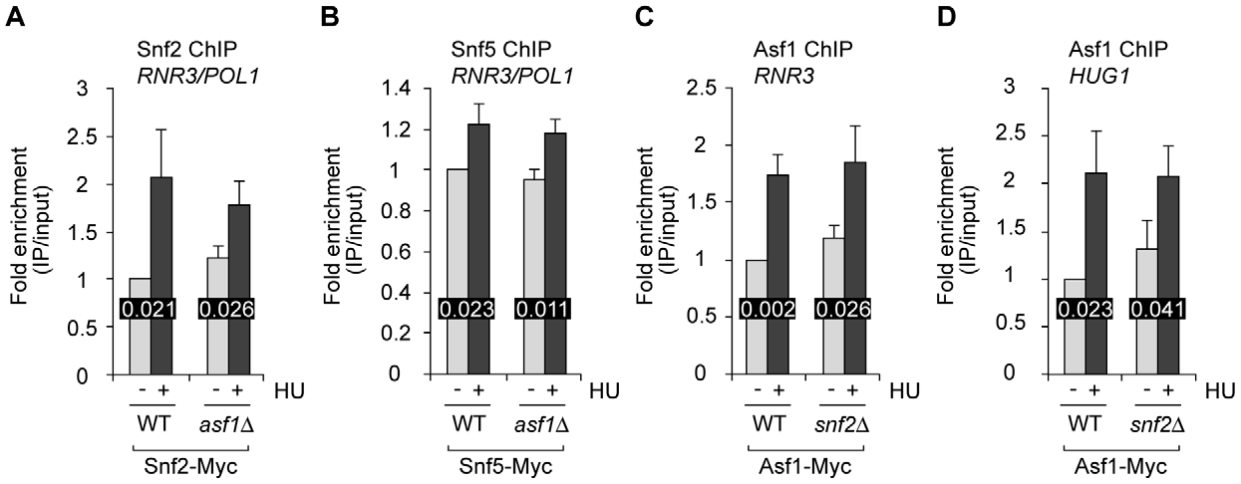
Asf1 and SWI/SNF are both recruited to the promoters of DDR genes during replication stress. A. ChIP analysis of Snf2 recruitment to the promoter of *RNR3* in response to HU. In this and all other panels of Figure 2, P values obtained by statistical analysis (Student's t-test) are shown for comparisons of HU-treated and untreated cells. B. ChIP analysis of Snf5 recruitment to the promoter of *RNR3* in response to HU. In A and B, normalization takes into account SWI/SNF subunit cross-linking in the ORF of *POL1*; occupancy in untreated wild type cells is set to 1. C, D. ChIP analysis of Asf1 recruitment to the promoters of DDR genes. Immunoprecipitated DNA was normalised to input DNA and the signal obtained in untreated wild type cells was set to 1. In A-D HU treatment was for one hour. All PCR reactions were performed in triplicate to obtain the data points reported in the graphs.

SWI/SNF contributes directly to derepression of *RNR3* in cells treated with MMS to induce genotoxic stress [30]: derepression is dampened in *snf2Δ* cells, and Snf2 recruitment to *RNR3* can be detected by chromatin immunoprecipitation (ChIP) [30], [35]. We similarly find that *RNR3* derepression in cells treated with HU requires *SNF2* (Figure 1C) and is associated with Snf2-myc recruitment to the promoter of *RNR3* (Figure 2A – WT). Therefore, the dependence of *RNR3* and *HUG1* derepression on *SNF2* (Figure 1B, C) likely reflects a direct role for SWI/SNF in chromatin remodelling that favours derepression when the replication stress checkpoint is triggered by depletion of dNTPs.

At *PHO5*, recruitment of SWI/SNF under low phosphate conditions depends on Asf1 [28]. Similarly, SWI/SNF association with the URS2 element of the *HO* promoter depends on Asf1 [25]. Based on this evidence, we tested whether Snf2 recruitment to *RNR3* in HU-treated cells is dependent on *ASF1*. As shown in Figure 2A (*asf1Δ* bars), deletion of *ASF1* does not have a strong effect on Snf2 recruitment to *RNR3* under conditions of replication stress. We next examined the association of another SWI/SNF subunit, Snf5, with the promoter of *RNR3* in wild type and *asf1Δ* cells. Although Snf5 was less readily detected by ChIP than Snf2 (see Materials and Methods), it appears that deletion of *ASF1* has little effect on Snf5 cross-linking to *RNR3* (Figure 2B). Like SWI/SNF, Asf1 occupancy at the promoters of *RNR3* and *HUG1* increases after treatment with HU or MMS [26]. This increase is readily detected in HU-treated wild type cells by ChIP (Figure 2C and D, WT bars). As shown in Figures 2C and D, Asf1 recruitment to *RNR3* and *HUG1* in response to HU is unaffected by deletion of *SNF2* (compare WT to *snf2Δ* bars). Collectively, our results support the hypothesis that Asf1 and SWI/SNF can be independently recruited to the promoters of DNA damage response genes during replication stress induced by treatment of cells with HU.

### Histone H3 lysine 56 acetylation is not perturbed in cells lacking *SNF2*

**Figure 3:**
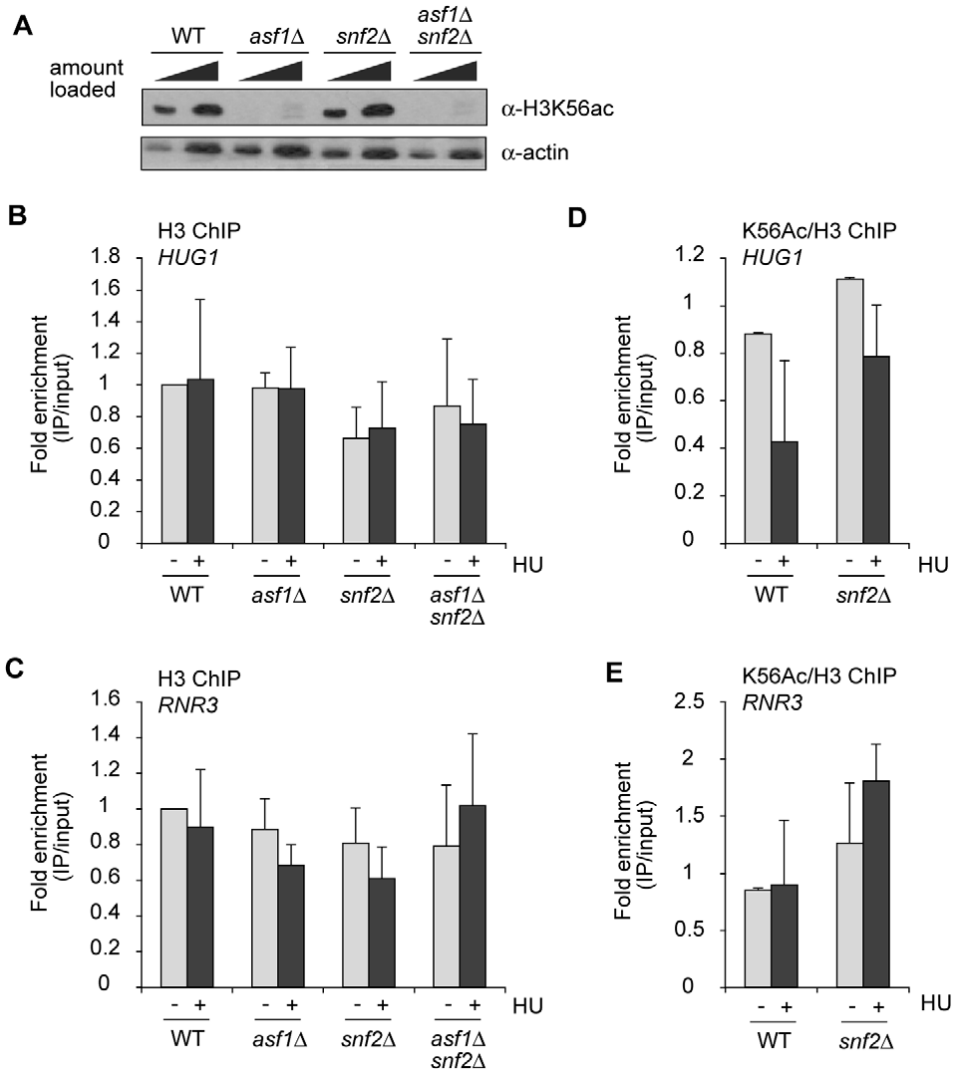
Regulation of H3K56ac is similar in wild type and *snf2Δ* cells. A. Bulk expression of H3K56ac analyzed by immunoblotting. Actin is the loading control. B, C. ChIP analysis of H3 occupancy at DDR gene promoters in response to HU (one hour). Immunoprecipitated DNA was normalised to input DNA and signal obtained in untreated wild type cells was set to 1. D, E. ChIP analysis of H3K56ac occupancy at DDR gene promoters in the indicated strains grown as in B and C. Normalization of the data is described in the methods section. In B-E, error bars show standard deviation between three biological replicates.

Asf1 promotes transcriptional derepression of the DDR genes by a mechanism that depends on acetylation of K56 in the histone fold domain of H3 [26]. In this pathway, Asf1 functions by stimulating the lysine acetyltransferase activity of Rtt109, the major enzyme in yeast capable of acetylating H3 at K56. These findings prompted us to test if there is functional interplay between Asf1 and SWI/SNF at the level of H3K56 acetylation. To determine whether Asf1 and SWI/SNF might work together in a pathway that regulates H3K56 acetylation, we first compared bulk H3K56 acetylation in wild type, *asf1Δ*, *snf2Δ*, and *asf1Δ snf2Δ* cells grown in rich medium. As expected [36]-[38], H3K56 acetylation could not be detected in *ASF1* null mutants (*asf1Δ* and *asf1Δ snf2Δ* in Figure 3A). Furthermore, H3K56 acetylation was similar in wild type and *snf2Δ* cells. Therefore, under normal conditions, *SNF2* is not important in otherwise wild type cells for bulk regulation of H3K56 acetylation.

We next used ChIP to determine whether SWI/SNF affects H3K56 acetylation at individual DDR gene promoters in normally cycling or HU-treated cells. H3K56ac cross-linking was normalized to H3 cross-linking, which did not vary substantially between strains or experimental conditions (Figure 3B, C; normalization also took into account the background signal due to non-specific binding of the anti-H3K56ac antibody – see reference 26). At *HUG1*, HU treatment is associated with a trend towards lower H3K56 acetylation in wild type cells (WT in Figure 3D). At *RNR3*, on the other hand, there is no effect of HU on promoter acetylation of H3K56 (WT in Figure 3E). In view of the variability associated with the *HUG1* measurements in HU-treated cells, and the results obtained for *RNR3*, we conclude that in wild type cells H3K56 acetylation does not consistently increase or decrease at DDR genes in response to HU treatment.

We compared H3K56ac in wild type and *snf2Δ* cells in order to determine if SWI/SNF affects H3K56ac metabolism at DDR gene promoters. These experiments revealed little effect of *SNF2* deletion on H3K56ac in the promoters of *HUG1* and *RNR3* in normally cycling or HU-treated cells (*snf2Δ* bars in Figure 3D, E). These findings suggest that the metabolism of H3K56ac at DDR gene promoters does not involve the Snf2 subunit of SWI/SNF. Overall, we conclude that Asf1 and SWI/SNF are not components of a linear pathway that affects H3K56 acetylation in the promoters of DDR genes.

### *ASF1* interacts genetically with *SNF2* and *SNF5*

**Figure 4:**
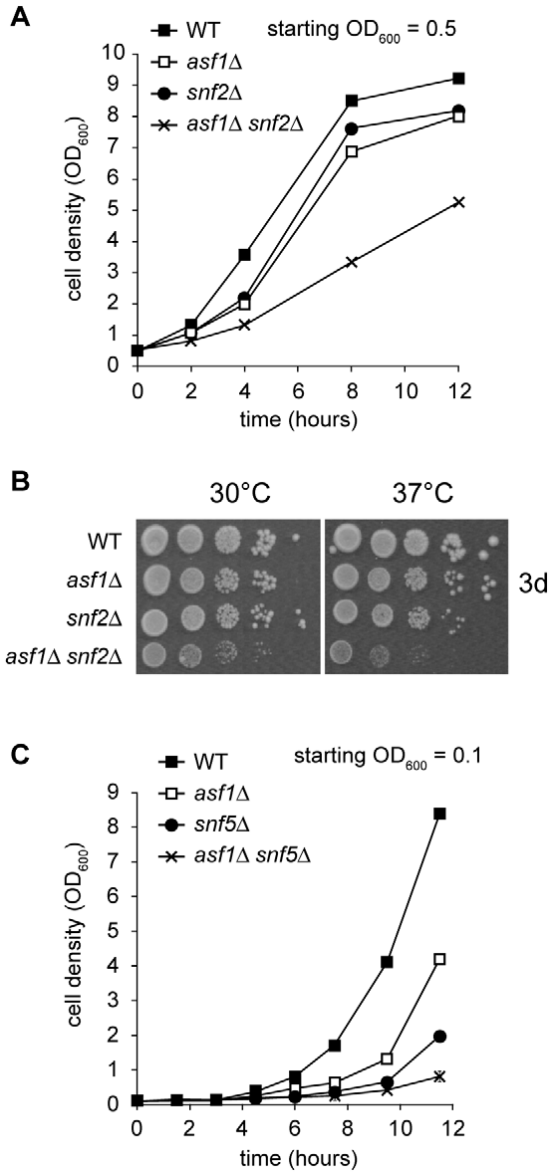
*ASF1* shows synthetic sick interactions with components of the SWI/SNF complex. A. *asf1Δ snf2Δ* double mutants are slower-growing than either single mutant. B. *asf1Δ snf2Δ* double mutants are slightly temperature-sensitive. Ten-fold serial dilutions of early log phase cells were photographed after 3 days of growth. C. *asf1Δ snf5Δ* double mutants are slower-growing than either single mutant. Averages of three independent *asf1Δ snf5Δ* isolates are shown, with error bars (under data point icons).

To explore why deletion of *ASF1* and *SNF2* has an additive effect on regulation of the DDR genes in cells treated with HU, we analyzed the proliferation and DDR signalling phenotypes of Asf1- and SWI/SNF-deficient strains. This work built on a synthetic genetic array analysis which uncovered a potential interaction between *ASF1* and *SNF2* (data not shown) [39]. To confirm this interaction, the growth rate of an *asf1Δ snf2Δ* haploid strain generated using one-step gene replacement was compared to the growth rates of the *asf1Δ* and *snf2Δ* single mutants under optimal culture conditions (rich medium with glucose at 30°C). The *asf1Δ* and *snf2Δ* single mutants grew at similar rates and both were slower-growing than the congenic wild type strain; the *asf1Δ snf2Δ* double mutant, however, was slower-growing than either single mutant (Figure 4A, left panel of Figure 4B). Therefore, there is a ‘synthetic sick’ genetic interaction between *ASF1* and *SNF2*. Consistent with this interpretation, an *asf1Δ snf2Δ* double mutant was more compromised for growth at 37°C than either single mutant, although *snf2Δ* cells displayed a slight growth defect at 37°C (Figure 4B). To determine whether *ASF1* also interacts genetically with other components of the SWI/SNF complex, an *asf1Δ snf5Δ* double mutant strain was generated. The *asf1Δ snf5Δ* double mutant grew slower than either of the corresponding single mutants (Figure 4C). We conclude that combined deletion of *ASF1* and SWI/SNF subunits results in synthetic growth defects.

### Separate and overlapping functions of Asf1 and SWI/SNF in cellular responses to structural perturbation of DNA

**Figure 5:**
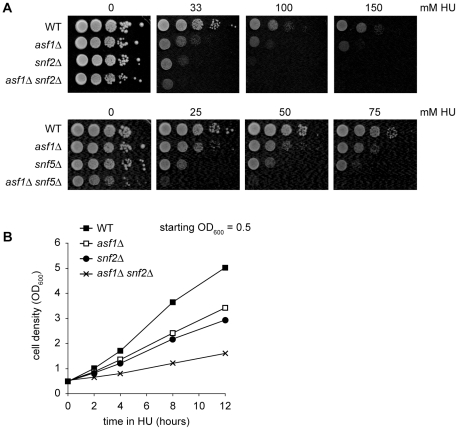
Asf1 and SWI/SNF contribute to survival under conditions of replication stress triggered by HU. A. *asf1Δ snf2Δ* (upper panel) and *asf1Δ snf5Δ* (lower panel) double mutants are sensitive to HU. Ten-fold serial dilutions of early log phase cells were photographed after 4 days of growth at 30°C. B. *asf1Δ snf2Δ* double mutants are slow-growing in liquid medium containing 0.2 M HU.

We extended the search for functional interactions between Asf1 and SWI/SNF by comparing the ability of cells lacking both Asf1 and a component of the SWI/SNF complex (Snf2 or Snf5) to grow after UV irradiation, or in the presence of HU or MMS; under all these growth conditions, cells must cope with elevated structural perturbation of DNA. Consistent with shared functions for Asf1 and SWI/SNF in protecting against replication stress, *asf1Δ*, *snf2Δ* and *snf5Δ* single mutants were all more sensitive to HU than wild type cells [16], [40] (Figure 5A). Furthermore, compared to the matching single mutants, *asf1Δ snf2Δ* and *asf1Δ snf5Δ* double mutants were hypersensitive to HU (Figure 5A). The proliferation phenotypes observed for wild type, *asf1Δ*, *snf2Δ* and *asf1Δ snf2Δ* strains in plating assays were readily apparent in liquid culture (Figure 5B). Therefore, Asf1 and SWI/SNF function in separate pathways to promote cellular resistance to replication stress caused by dNTP depletion.

**Figure 6:**
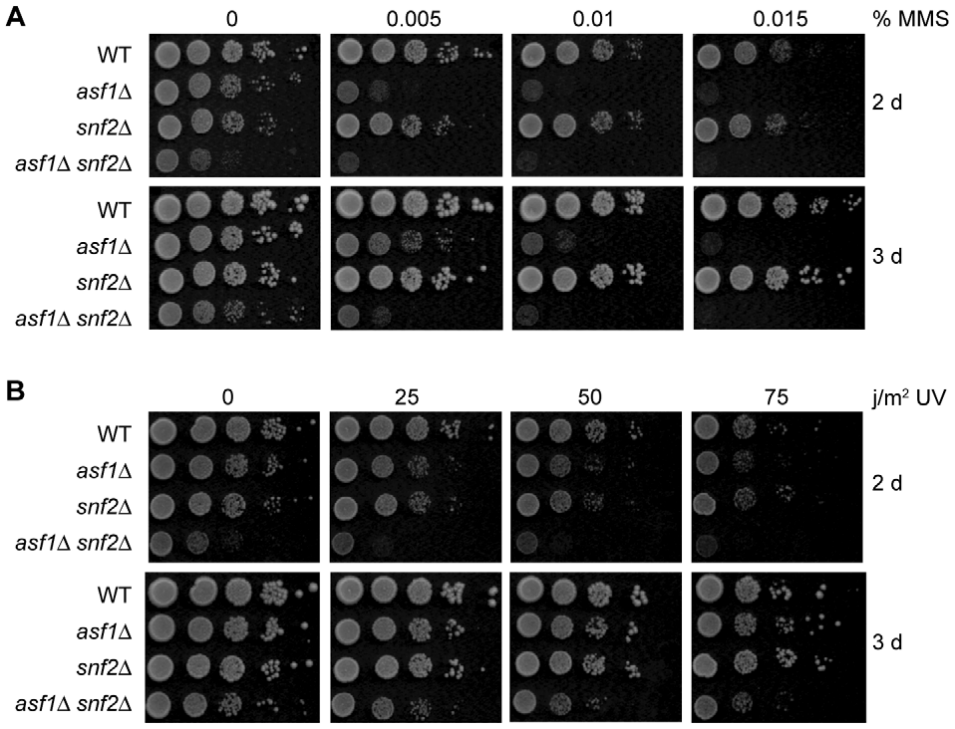
*asf1Δ snf2Δ* mutants are sensitive to genotoxins that cause chemical modification of DNA. A. *asf1Δ snf2Δ* double mutants are sensitive to MMS. Ten-fold serial dilutions of early log phase cells were spotted onto rich medium with or without MMS and grown at 30°C. Photographs were taken after 2 and 3 days. B. *asf1Δ snf2Δ* double mutants are sensitive to UV irradiation. Ten-fold serial dilutions of cells were spotted onto rich medium and irradiated or not. Plates were incubated in the dark at 30°C and photographed as in (A).

MMS and UV light can elicit DNA structure perturbation responses that are not triggered by HU. For example, the mechanisms used to repair the primary DNA lesions caused by MMS and UV light [41] are not activated by fork stalling in HU-treated cells. The genetic requirements for optimal proliferation in the face of exposure to HU therefore may not be the same as those for optimal proliferation under conditions of genotoxic stress induced by MMS and UV light. Indeed, *asf1Δ* cells do not have the same MMS sensitivity as *snf2Δ* cells: cells lacking *ASF1* are sensitive to MMS (as previously reported) [40], while cells lacking *SNF2* are not (Figure 6A). The most straightforward interpretation of these results is that Asf1, but not Snf2, is in a pathway that confers resistance to MMS. Interestingly, the sensitivity of *asf1Δ* cells to MMS is enhanced by deletion of *SNF2* (Figure 6A). This finding suggests that normal cellular functions promoted by Snf2 are important for survival of exposure to MMS when Asf1 is absent from the cell. Although UV sensitivity was assessed by monitoring proliferation after a treatment pulse rather than continuous exposure, and killing of wild type cells was only modest at the UV dose used, the sensitivity profile of the single and double mutants to UV light (Figure 6B) was found to be much the same as the sensitivity profile to MMS (Figure 6A). Collectively, the results of the MMS and UV sensitivity assays support the proposal that Snf2 protects *asf1Δ* cells from the adverse effects of chemical modification of DNA by exogenous genotoxins.

### Contributions of Asf1 and Snf2 to checkpoint control in HU-treated cells

**Figure 7:**
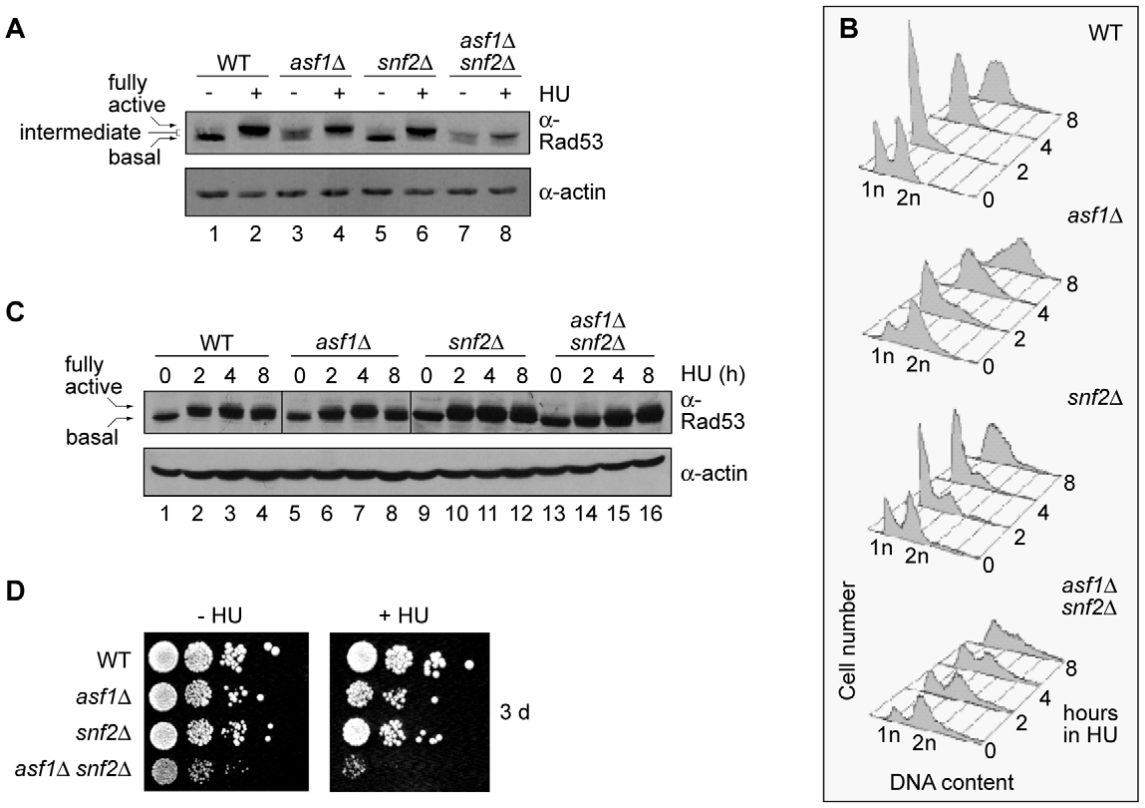
Asf1 and Snf2 have partially overlapping functions in cell cycle and checkpoint control. A. Rad53 activation is impaired in *asf1Δ snf2Δ* double mutants. Cells were grown in the presence or absence of 0.2 M HU for two hours. Actin is the loading control. The apparent lower expression of Rad53 in the double mutant was not reproducible – see Figure 7B. B. *asf1Δ snf2Δ* mutants fail to fully arrest in S phase in response to HU. DNA content of normally cycling cells (time 0) and cells grown in the presence of 0.2 M HU. C. Rad53 activation is delayed in *asf1Δ snf2Δ* double mutant cells. Cells from the cultures assayed in B were processed for analysis of Rad53 modification state. Actin is the loading control. D. Recovery from replication stress is compromised in *asf1Δ snf2Δ* mutants. Ten-fold serial dilutions of early log phase cells were grown in YPD or YPD + 0.2 M HU for 24 hours. Aliquots of these cultures were then diluted to 1×10^6^ cells/mL and spotted onto rich solid medium. Cells were grown at 30°C and photographs were taken after 2 and 3 days.

The synthetic sick phenotype of *asf1Δ snf2Δ* cells on HU suggests that the individual mutations confer HU sensitivity by different mechanisms. This proposition is supported by the results of our studies of the checkpoint signaling and cell cycle responses of the mutants to HU. Deletion of *ASF1* is associated with partial activation of the DNA damage checkpoint signaling pathway, in which Rad53 is a critical transducer kinase [42], [43]. A hallmark of Rad53 activation is its shift towards a more phosphorylated state; this shift can be detected by SDS-PAGE and anti-Rad53 immunoblotting. We tested whether expression of Snf2 affects Rad53 activation in wild type and/or *asf1Δ* cells cultured under normal conditions, and under standard conditions used to study replication stress triggered by depletion of dNTPs (0.2 M HU, 2 hours). Figure 7A shows that on its own, the absence of *SNF2* has no effect on Rad53 phosphorylation state under normal culture conditions (compare lanes 1, 5). Rad53 is also activated normally in the *snf2Δ* mutant when it is cultured for two hours in 0.2 M HU (Figure 7A - compare lanes 2, 6). While deletion of *SNF2* has no obvious effect on Rad53 activation when Asf1 is present in the cell, it does impact on Rad53 activation in cells which do not express Asf1. Specifically, the Rad53 mobility change that corresponds to the shift from intermediate to full activation in *asf1Δ* cells (Figure 7A - lanes 3, 4) is dampened in the *asf1Δ snf2Δ* double mutant (Figure 7A, lanes 7, 8), even though activation is normal in cells lacking only *ASF1* or only *SNF2* (Figure 7A - compare lanes 2, 4, 6).

We hypothesized that the low proliferation rate of *asf1Δ snf2Δ* cells (Figure 4, 5B) accounts for their delayed activation of Rad53 in response to replication stress. More specifically, we envisaged that the Rad53 activation defect of *asf1Δ snf2Δ* cells grown in the presence of HU reflects their abnormally slow accumulation in S phase (the time when DNA structure perturbations that activate the replication stress checkpoint are generated) [44]. Indeed, S phase cells predominate in wild type, *asf1Δ* and *snf2Δ* cultures at two hours after HU addition, but are in the minority in *asf1Δ snf2Δ* cultures (Figure 7B). A further prediction of the slow proliferation hypothesis is that as the proportion of S phase cells in *asf1Δ snf2Δ* cultures increases, so should the amount of activated Rad53. To test this prediction, Rad53 activation was monitored in cells that were treated with HU for up to eight hours. Figure 7B shows that the proportion of S phase cells in the *asf1Δ snf2Δ* culture increases throughout the time course of this experiment. And after eight hours in HU, Rad53 is activated to near wild type levels in the *asf1Δ snf2Δ* mutant (Figure 7C, compare lanes 13-16). Altogether, these results support the notion that Rad53 activation is delayed in HU-treated *asf1Δ snf2Δ* double mutants because they are defective for S phase progression and are therefore less able to generate the abnormal DNA structures which trigger the replication stress checkpoint.

While all individual cells in an *asf1Δ snf2Δ* culture likely contain the wild type amount of active Rad53 after eight hours in HU, their overall physiological response to long-term exposure to this replication inhibitor is not normal. Specifically, the plating efficiency of *asf1Δ snf2Δ* cells after one day culture in HU is substantially lower than the plating efficiency of either single mutant (Figure 7D). We conclude that *asf1Δ snf2Δ* mutants are unable to recover from extended replication stress despite their ability to eventually activate Rad53.

## Discussion

The evidence that *PHO5* and *HO* depend on Asf1 and SWI/SNF for induction, and that a SWI/SNF recruitment step at these genes depends on Asf1, strongly suggests an important role for Asf1 in SWI/SNF functions that affect transcription [25], [28], [29]. However, the alternative possibility — that Asf1-stimulation of SWI/SNF occupancy is unimportant for events that lead to transcription initiation — is not excluded by such correlations because the separate steps of SWI/SNF association with these genes do not have the same requirement for Asf1. Indeed, based on genetic studies of the requirements for Snf2 recruitment to *PHO5* [28], and the functions of *ASF1* and *SNF2* in its induction [29], Adkins et al. have speculated that stimulation of SWI/SNF recruitment by Asf1 is not necessary for SWI/SNF regulation of *PHO5* transcription [28]. Our findings reveal that the DDR genes in yeast, like *PHO5*, can be induced by a mechanism that requires Asf1 and SWI/SNF but does not depend on their direct functional interaction. Thus, although Asf1 and SWI/SNF can occur together in the same protein complex (Figure 1A), they can be independently recruited to the DDR genes and evidently modulate their derepression by unlinked mechanisms.

The significance of the presence of Snf2 in the complex obtained by tandem affinity purification of Asf1 has yet to be determined. Since SWI/SNF recruitment to *PHO5* and the URS2 element of the *HO* promoter requires Asf1 [28], [45], one possibility is that a complex that includes Asf1 and Snf2 forms in the course of activation of such genes. The possibility that this complex assembles only on DNA would not preclude its appearance in the nucleoplasm. For example, engagement of the mechanisms that shut off genes such as *HO* and *PHO5* might involve promoter eviction of a protein assemblage containing Asf1 and SWI/SNF.

The synthetic effect of deletion of *ASF1* and *SNF2* on derepression of the DDR genes is reminiscent of the synthetic effect of these mutations on *PHO5* induction in low phosphate medium [29]. Korber et al. [29] outlined a highly plausible model to account for the synthetic effect of *asf1Δ* and *snf2Δ* on *PHO5* regulation. In one version of this model, *PHO5* induction is partly inhibited in *asf1Δ* cells because, although an alternative chaperone functions in concert with SWI/SNF to promote chromatin reconfiguration that favours initiation, it does so by a reaction that is less efficient than the one involving Asf1 and SWI/SNF. Similarly, in a strain lacking Snf2, the chromatin reconfiguration that favours *PHO5* transcription involves Asf1 and a different remodeler; again, this alternative pathway is presumed to be less efficient than the one involving Asf1 and SWI/SNF. We do not rule out similar mechanistic contributions to the regulation of the DDR genes in *asf1Δ* and *snf2Δ* mutants. However, our evidence supports an alternative explanation for the synthetic effects of *asf1Δ* and *snf2Δ* on DDR gene regulation which does not invoke the participation of alternative pathways of chromatin remodelling. We find that combined deletion of *ASF1* and *SNF2* is associated with a pronounced defect in cell cycle progression in the presence of HU (Figure 7B). Since HU-treated cells must proceed into S phase in order to activate the checkpoint that triggers derepression of the DDR genes, the *asf1Δ snf2Δ* double mutant is expected to be defective for checkpoint engagement. Analysis of Rad53 activation suggests this to be the case (Figure 7C). It follows that *asf1Δ snf2Δ* cells are impaired for derepression of the DDR genes because of a cell cycle defect that hampers engagement of replication stress checkpoint signalling.

## Materials and Methods

### Strains, plasmids and liquid media

Strains used are listed in Table 1; all are derived from BY4741. Single deletion mutants, from the *S. cerevisiae* haploid nonessential gene deletion library [46], were verified to be correct by PCR using multiple primer sets. Chromosomal mutations were generated by one-step integration using PCR products obtained from previously described plasmids [47]-[50]. Addition of sequences encoding the 13-Myc and 3-HA epitope tags, and the TAP tag, was verified by PCR using three primer sets: primers flanking the target gene (upstream, downstream) plus primers specific to the tag, and a primer flanking the target gene plus a primer specific for the *HIS* selection marker. All selection media were prepared as described previously, and standard genetic methods for transformations were used throughout [51]. Batch-culture for all the experiments shown was performed in YPD: yeast extract (1%) – bactopeptone (2%) – dextrose (2%).

**Table 1:** Strains used in this study.

Strain	Genotype	Reference
BY4741	*MAT*a *his3Δ1 leuΔ0 met15Δ0 ura3Δ0*	[55]
Y2454	*MAT*a *mfa1Δ::MFA1pr-HIS3 can1Δ ura3Δ0*	[56]
*snf2Δ*	*snf2Δ::kanMX*	[46]
*asf1Δ*	*asf1Δ::kanMX*	[46]
*asf1Δ*	*asf1Δ::NAT* (in Y2454)	This study
*SNF2-HA*	*SNF2-3HA::kanMX*	This study
*SNF2-HA ASF1-TAP*	*SNF2-3HA::kanMX ASF1-TAP::HIS*	This study
SY1	*SNF2-13MYC::HIS3*	[57]
*asf1Δ SNF2-MYC*	*asf1Δ::NAT* in SY1	This study
*SNF5-MYC*	*SNF5-13MYC::HIS3*	This study
*asf1Δ SNF5-MYC*	*asf1Δ::kanMX SNF5-13MYC::HIS3*	This study
*snf2Δ ASF1-MYC*	*snf2Δ::kanMX ASF1-13MYC::HIS3*	This study
*asf1Δ snf2Δ*	*asf1Δ::NAT snf2Δ::kanMX*	This study
*asf1Δ snf5Δ*	*asf1Δ::NAT snf5Δ::kanMX*	This study

### Spotting assays

Except where noted (Figure 7D), cells were grown to early log phase in YPD, diluted to 1×10^7^ cells/mL and 10-fold serial dilutions were spotted onto solid YPD medium.

### Flow cytometry analysis of DNA content

Fixed cells were stained with propidium iodide, sonicated and analyzed using a FACScan flow cytometer (Becton-Dickinson) [52].

### RNA isolation and analysis

Total RNA was isolated by hot phenol extraction [53] from cells grown as described in the relevant figure legends. DNA probes for Northern blotting were prepared by random primed labelling of PCR products (sequences available upon request). Phosphorimaging was used for data collection, and ImageQuant TL software was used to quantitate signals including the *SCR1* loading control.

### Immunoblotting

Total proteins were prepared by trichloroacetic acid precipitation [52]. Identical cell equivalents of protein were compared between samples. Antibodies were as follows: α-Rad53 (yC-19, Santa Cruz #sc-6749), α-H3 (Abcam #ab1791), α-actin (Millipore #MAB1501), α-myc (Millipore #9E10), α-H3K56ac (Upstate #07-677), α-HA (Roche, #12CA5) and α-CBP (Upstate #07-482).

### Tandem Affinity Purification

Tandem affinity purification of protein complexes was performed essentially as described [31].

### Chromatin Immunoprecipitation

Chromatin immunoprecipitation was performed according to Minard et al., including wash-out of hydroxyurea prior to fixation [54]. Statistical significance was assessed by applying Student's unpaired (independent) two-tailed t-test for independent samples (three or more) of each strain under consideration.
